# Improving labial and nasal outcome in a secondary bilateral cleft lip and palate patient using the Mulliken method of repair

**DOI:** 10.1016/j.jpra.2024.02.008

**Published:** 2024-02-15

**Authors:** Tristan de Chalain, Daryl Crimmins

**Affiliations:** a Aesthetic and Reconstructive Plastic Surgery, Level 2, OneHealth Building, 122 Remuera Road, Remuera, Auckland 1050, New Zealand; b F7G8 Ltd, Christchurch, New Zealand

**Keywords:** Bilateral cleft lip and palate, Mulliken repair, Rhinoplasty, Multidisciplinary care

## Introduction

Approximately 0.10% or 1/940 live births result in children with cleft lip and palate congenital anomalies.^1^ These children require multidisciplinary care and reconstructive procedures throughout childhood and adolescence to support otolaryngologic improvement, speech and dental development alongside psychosocial well-being to mitigate negative impacts and barriers to social integration, given the functional and visible nature of the condition.^2^^,^^3^

The etiology of cleft lip and palate abnormalities is still poorly understood. Most researchers invoke multifactorial interplay between genetic and environmental factors such as folic acid antagonists that act as dihydrofolate reductase inhibitors inhibiting normal molecular growth cascade patterns that lead to a coalescence between the medial nasal process and maxillary prominence at around four to six weeks gestation.^2^^,^^4^

Over the past decades, the older, staged operations involving techniques such as a Manchester straight line repair for primary cleft lip and palate have been superseded by synchronous correction of the bilateral labial cleft, gums, and nasal deformity using techniques pioneered by Mulliken, Noordhof and Cutting.^5^ These techniques, which are now widely used for primary cleft repairs are also suitable for secondary use in improving results in older patients whose primary repairs were carried out using older, less satisfactory techniques. This short communication describes the successful, secondary use of the Mulliken technique to revise an adult bilateral cleft lip and palate which had undergone a primary Manchester repair in infancy.

## Case background

A 41-year-old man, born with a bilateral cleft lip and palate, was initially under the care of a NZ regional cleft unit. The primary surgery, a Manchester repair, was carried out at the cleft unit. Primary rhinoplasty was performed at the time of Alveolar bone grafting to correct the alveolar cleft bony defect at age 14. This approach, standard at the time, has become outdated and clearly caused characteristic and unsatisfactory effects on the nasal cartilaginous and soft tissues, especially the columella, in this patient. Attempts to revise the nasal structure under both the public cleft unit and private outpatient clinic setting failed to adequately address the negative effects of the primary and ensuing, staged surgery. Subsequently, having moved cities, the patient came forward for review at another regional Hospital Cleft unit as a young adult. His-concerns, which were both functional and aesthetic, were acknowledged but were not attended to as they were deemed to be of low clinical priority.

Over the last 17 years clinical oversight has been provided in the private practice setting. We have operated on several occasions to address secondary complications affecting his cleft lip and palate as well as residual problems relating to his nose. To date he has had reconstructive rhinoplasties with rib and auricular cartilage grafts and two lip revisions as well as two attempts to close palatal fistulae.

## Discussion

**Figure 1:**
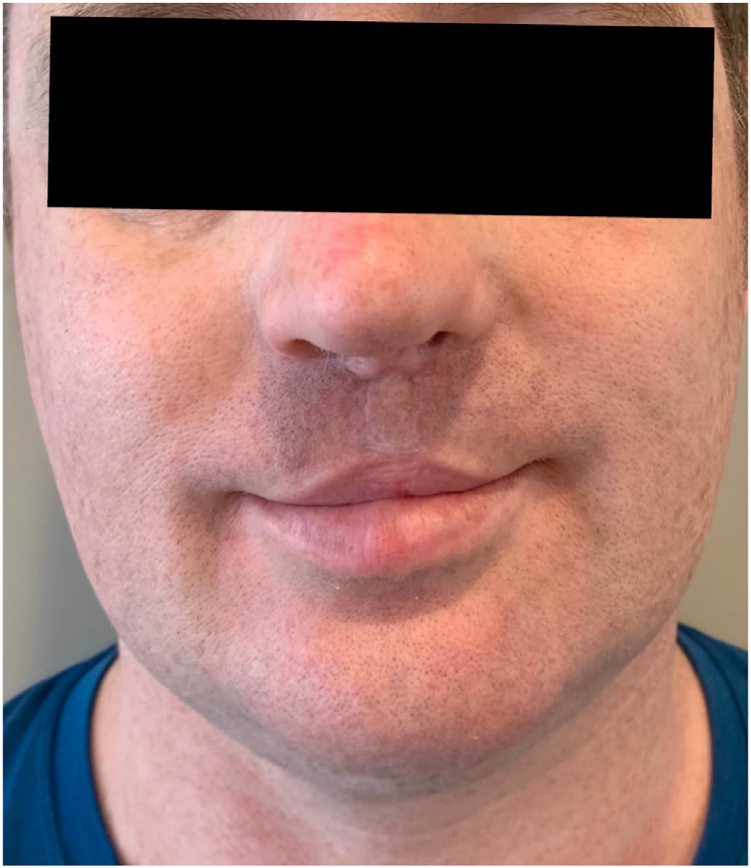
Bilateral cleft lip nasal deformity. Patient at 41 years following Mulliken Repair and reconstructive rhinoplasty using both rib cartilage and primary conchal cartilage grafts from both left and right ears to stabilise the hypoplastic left nostril.

**Figure 2:**
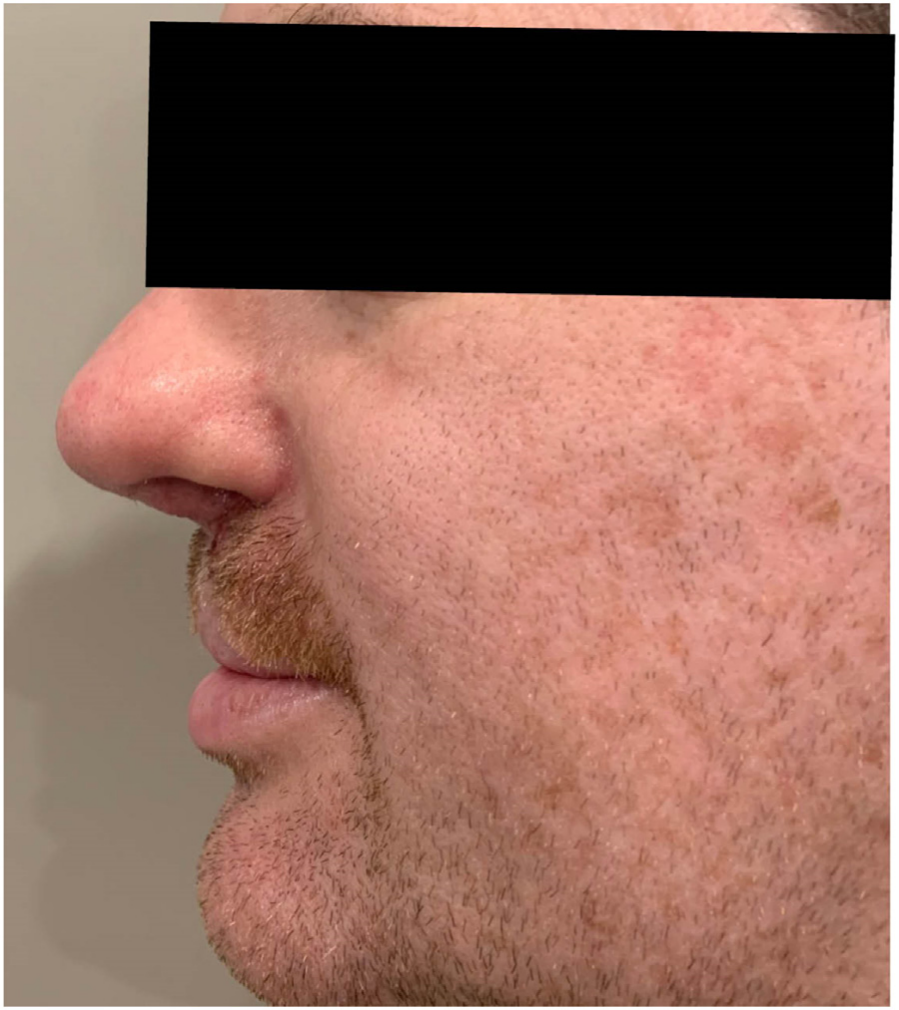
Nasal projection post adult cleft rhinoplasty following rib cartilage graft manipulation of the bony pyramid and nasal septum alongside introduction of primary iliac cartilage grafts to improve both structure and function.

The Mulliken repair corrects both the cleft lip and nasal deformity simultaneously and places a strong emphasis on facial growth in children as a key measure for good cleft care rather than undue reliance on the now outdated strategy of secondary nasal correction in patients with repaired cleft lip and cleft palate.^2^^,^^5^ Following the reconstructive rhinoplasty and Mulliken cleft lip repair this patient's nasal length, nasal tip projection, columellar length, and upper lip shape were significantly improved (Figure 1). Nasal tip protrusion, nasal width, upper lip height, and vermilion-mucosal height were also additionally amended (Figure 2).

Current guidelines recommend against any primary “tip rhinoplasty” operations during puberty, when the nose is growing and changing rapidly. Cleft rhinoplasty is now a secondary procedure undertaken after skeletal maturity at 17 years and over for men.^5^ Adult patients like this one, who underwent the Manchester cleft lip repair, encounter significant clinical and aesthetic complications particularly with regard to the philtrum inexorably widening, as *orbicularis oris* is not connected across the midline. The palatal fistula which remains is a significant challenge in the cleft population. This may or may not have functional consequences in the future. New technologies will be required to address difficult palatal fistulas and close this gap in treatment options for cleft lip and palate patients.

## Conclusion

To our knowledge this is the first described case that spans 43 years illustrating the complexities of bilateral cleft lip and palate care and details how techniques like that proposed by John Mulliken have improved the results in adult bilateral cleft patients who underwent, older, less satisfactory repairs.

## Disclosure

This research did not receive any specific grant from funding agencies in the public, commercial or not-for-profit sectors.

## Patient consent

Patient have given informed consent to the publication of images and/or data.

## Funding declaration

The authors received no financial support for the research, authorship, and/or publication of this article

## Funding

None.

## Ethical approval

Not required.

## Declaration of competing interest

The authors declare that they have no known competing financial interests or personal relationships that could have appeared to influence the work reported in this paper.
